# Supplementary material for: Implications of Podoplanin Overexpression in the Malignant Transformation of Oral Potentially Malignant Disorders: A Systematic Review and Meta-Analysis
Source: Cancers (Basel). 2025 Oct 28;17(21):3448. doi: 10.3390/cancers17213448 (PMC12609328; doi:10.3390/cancers17213448)
Supplement: Supplementary file 1 [file cancers-17-03448-s001.zip › cancers-3942480-supplementary.pdf]

Supplementary information to the manuscript

Implications of podoplanin overexpression in the malignant transformation of oral potentially malignant disorders: a systematic review and meta-analysis

Table of contents

1. Search strategy....3

2. Descriptive characteristics of the study sample....4

3. Meta-analyses on the association between the podoplanin overexpression and the malignant transformation risk of OPMDs 6

4. Analysis of small-study effects. 15

5. Sensitivity analysis 16

6. List of excluded studies with reasons 17

1. Search strategy

Table S1. Search strategy for each database, number of results, and execution date.

Database	Query/Search Strategy	Results/ Items found	Search time limits
MEDLINE	("podoplanin"[All Fields] OR "PDPN"[All Fields] OR "T1A-2"[All Fields] OR "Gp38"[All Fields] OR "aggrus"[All Fields] OR "GP40"[All Fields] OR "PA2.26"[All Fields] OR "D2-40"[All Fields] OR "lung type I cell membrane associated glycoprotein"[All Fields]) AND ("mouth"[MeSH Terms] OR "mouth"[All Fields] OR "oral"[All Fields] OR oropharyn*[All Fields]) AND ("carcinoma, squamous cell"[MeSH Terms] OR ("carcinoma"[All Fields] AND "squamous"[All Fields] AND "cell"[All Fields]) OR "squamous cell carcinoma"[All Fields] OR “dysplasia"[All Fields] OR “potentially malignant disorders”[All Fields] OR premalign*[All Fields] OR precancer*[All Fields] OR “leukoplakia”[All Fields] OR “erythroplakia”[All Fields] OR “lichen planus”[All Fields] OR “submucous fibrosis”[All Fields]) AND (“transformation”[All Fields] OR “progression”[All Fields] OR “risk”[All Fields])	99	Nov-2024
Embase	('podoplanin' OR 'PDPN' OR 'T1A-2' OR 'Gp38' OR 'aggrus' OR 'GP40' OR 'PA2.26' OR 'D2-40' OR 'lung type I cell membrane associated glycoprotein') AND ('squamous cell carcinoma'/exp OR 'squamous cell carcinoma' OR ('squamous' AND ('cell'/exp OR 'cell') AND ('carcinoma'/exp OR 'carcinoma')) OR 'dysplasia' OR 'potentially malignant disorders' OR 'premalign*' OR 'precancer'/exp OR 'precancer*' OR 'leukoplakia'/exp OR 'leukoplakia' OR 'erythroplakia' OR 'erythroplakia' OR 'lichen planus'/exp OR 'lichen planus' OR 'submucous fibrosis') AND ('transformation' OR 'progression' OR 'risk')	342	Nov-2024
Web of Science	TS=("podoplanin" OR "PDPN" OR "T1A-2" OR "Gp38" OR "aggrus" OR "GP40" OR "PA2.26" OR "D2-40" OR "lung type I cell membrane associated glycoprotein") AND TS=(mouth OR oral OR oropharyn*) AND TS=(“squamous cell carcinoma”) AND TS=(transformation OR progression OR risk)	113	Nov-2024
Scopus	TITLE-ABS-KEY(("podoplanin" OR "PDPN" OR "T1A-2" OR "Gp38" OR "aggrus" OR "GP40" OR "PA2.26" OR "D2-40" OR "lung type I cell membrane associated glycoprotein") AND (“mouth” OR “oral”) AND (“squamous cell carcinoma” OR neoplas* or “cancer”))	283	Nov-2024
Total			837

2. Table S2 .Characteristics of the included studies (n = 12)

Study (year)	Country	Publication language	Study design (recruitment period)	Follow up, m, mean±SD (range)	Patients with precancerous status and progression to cancer								Analysis of podoplanin protein expression				
					Sample Size, n	Sex, M(%)/F, n; age, y (mean±SD, range)	Tobacco, n (%)	Alcohol, n (%)	Affected oral subsites	OPMDs	Epithelial dysplasia (grade), n	Cancer development, n	Methods	Antibody (dilution, incubation time, temperature)	IHC patern	IHQ cutoff point (%)	Podoplanin positivity, n(%)
Alkan et al (2022)	Israel	English	Retrospective cohort (1995-2016)	86±nr (36-288)	34	M=nr F=nr nr ± nr	nr	nr	nr	Leukoplakia Proliferative verrucous leukoplakia	No ED:15 Missing:19	29	IHQ	Clone D2-40 (1:100, nr, nr)	nr	Labelling index (intensity)	24 (70.59)
Monteiro et al (2022)	Portugal	English	Retrospective cohort (1995-2006)	32.4 ± 29 (2-120)	52 (missing:13)	M=38 (73.08) F=14 (57.55±16.54) (20-88)	Current: 12 Former or never: 22 Missing: 17	Current:111 Former or never: 24 Missing: 17	Bm:12 Tongue:23 Gingiva:8 FOM:2 Palate:1 Lip:4 Others:2	Leukoplakia	No ED:nr ED: 52 (low grade:41 high grade:11)	6	IHQ	Clone D2-40 (1:150, nr, nr)	membrane	Labelling index (intensity x cell count)	5 (12.82)
Verma et al (2019)	India	English	Prospective cohort (nr)	nr±nr (6 -24)	60	M=53 (88,33) F=7 (52.57 ± 11.17)	Smoking/ chewing:57	nr	Bm:39 Tongue:8 Gingiva:3 Lip:10	Leukoplakia Erythroplakia	No ED:0 ED: 60 (mild:20 moderate:20 severe:20)	15	IHQ	Clone D2-40 (nr, 60 min, 37°)	membrane	1	45 (75.00)
D´Souza et al (2018)	India	English	Retrospective cohort (nr)	nr ± nr (nr-nr)	30	M=22 (73.33) F=8 (49.86±nr, 18-70)	Chewing :18 Smoking: 4 Gutkha chewing:1 Tobacco +gutkha: 3 Gutkha+smoking:1 Areca nut:2	Alcohol:1	Bm:27 Tongue:2 Gingiva:1	Leukoplakia	No ED:9 (mild:9 moderate:6 severe:5)	1	IHQ	Clone D2-40 (nr, nr, nr)	Cytoplasmic	≥1% of suprabasal cells	23 (76.67)
Habiba et al. (2017)	Japan	English	Retrospective cohort (2002-2012)	42.1±34.1 (6-125)	79	M=25 (32.00) F=54 (70±12)	nr	nr	Bm:21 Tongue:28 Gingiva:18 FOM:5 Other:7	Leukoplakia	No ED:0 ED: 79 (low grade:27 high grade:52)	37	IHQ	Clone D2-40 (1:100, overnight, 4°C)	Cytoplasmic-membrane	1	53 (67.09)
Zhang et al. (2017)	South Korea	English	Retrospective cohort (1994-2009)	Median=135±nr (55.2-278.4)	160	M=100 (62.50) F=60 (51.9±14.28)	nr	nr	Bm:44 Tongue:44 Gingiva:72	Leukoplakia	No ED:82 ED: 78 (Low grade:54 High grade:24)	22	IHQ	nr (1:100,nr,nr)	Cytoplasmic-membrane	1	37 (23.13)
Gao et al (2016)	China	English	Retrospective cohort (1993-2009)	nr ± nr (nr-nr)	52	M= 22 (42.31) F=30 (57.34±11.83, 40-86)	Never:32 Past and present:15 Unknown:5	Never:34 Past and present: 12 Unknown:6	Lip:52	Discoid lupus erythematosus	nr	8	IHQ	nr (1:150, nr, nr)	Cytoplasmic-membrane	≥1% of suprabasal cells	19 (36.54))
De Vicente et al (2013)	Spain	English	Retrospective cohort (2000-2005)	nr ± nr (nr-250)	58	M=31 (53.45) F=27 64.08 ± 12.7	Yes: 35 No: 23	Yes: 28 No: 30	nr	Leukoplakia	No ED:0 ED: 79 (mild:43 moderate:7 severe:8)	13	IHQ	Clone D2-40 (1:100, overnight, 4°C)	Cytoplasmic-membrane	≥1% of suprabasal cells	22 (37.93)
Feng et al (2012)	China	English	Retrospective cohort (1993-2009)	nr±nr (nr)	34	M=16 (47.06) F=18 (58.71±15.55) , 27-86)	nr	nr	Bm:9 Tongue:16 Gingiva:4 Palate:4 Other:1	Eritroplakia	No ED:0 ED: 34 (mild:4 moderate:16 severe:14)	17	IHQ	Clone D2-40 (1:150, nr, nr)	Cytoplasmic-membrane	≥1% of suprabasal cells	15 (44.11)
Kreppel et al (2012)	Germany	English	Retrospective cohort (2005-2007)	48.0 ± 15.8 (18.6-93.70)	60	M=32 (53.33) F=28 58.6 ± 16.7	Current: 21 Former: 11 Never: 28	Current:9 Ocassionally :22 More than once a week:8 Never:29	Bm:16 Tongue:6 Gingiva:12 FOM:9 Palate:17	Leukoplakia	No ED: 31 ED: 29 (SIN I: 8 SIN II: 12 SIN III: 9)	12	IHQ	Clone D2-40 (1:100, overnight, 4°C)	membrane	≥1% of suprabasal cells	41 (68.33)

**Study (year)**	**Country**	**Publication language**	**Study design (recruitment**	**Follow up, m,**	**Patients with precancerous status and progression to cancer**								**Analysis of podoplanin protein expression**				
Shi et al (2010)	China	English	Retrospective cohort (1978-2007)	61.2± nr (15.96-300)	119	M= 27 (22.69) F=92 (45.97±12.28, 9-74)	Never:83 Past and present:8 Missing:28	Never:81Past and present: 10 Missing:28	nr	Oral Lichen planus	No ED:99 ED: 20 (mild:11 moderate: nr severe: nr)	9	IHQ	Clone D2-40 (1:150, overnight, 4°C)	Cytoplasmic-membrane	≥1% of suprabasal cells	56 (47.06)
Kawaguchi et al. (2008)	USA	English	Prospective cohort (1994-2001)	90±nr (2.4-163.5)	150 (missing:18)	M=80 (53.33) F=70 (56.03±13.58, 23-90)	Current: 52 Former: 60 Never: 38	Current:85 Former:18 Never:46	nr	Leukoplakia	No ED: 101 ED: 49 (mild: 37 moderate: 10 severe: 2)	35	IHQ	Clone D2-40 (1:100, overnight, 4°C)	membrane	≥1% of suprabasal cells	56 (37.33)

Abbreviations: n, number; m, months; y, years; SD, standard deviation; nr, not reported; OPMD, oral potentially malignant disorder; ED, epithelial dysplasia; IHQ, immunohistochemistry.

3. Meta-analysis on the association between podoplanin overexpression and OPMDs malignant transformation risk

3.1 Subgroup analysis by geographical region

Figure S1. Forest plot graphically representing the stratified meta-analysis on the association between podoplanin overexpression and OPMDs malignant transformation risk by geographical region.

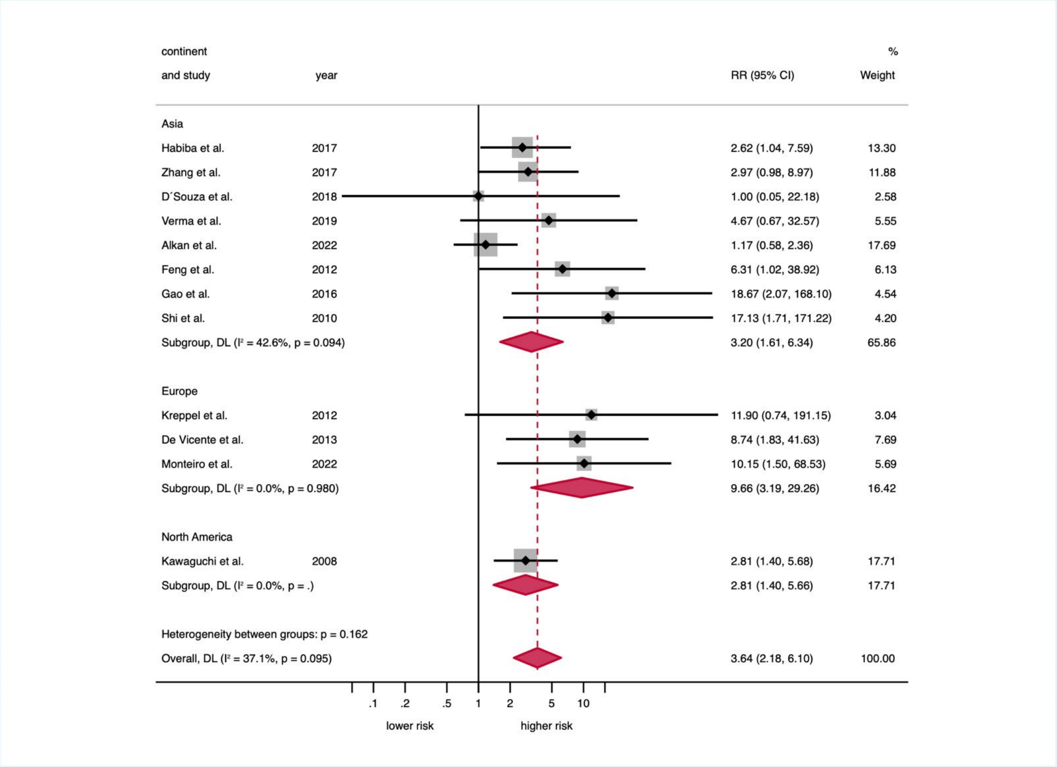

RR, relative risk; CI, confidence intervals, DerSimonian and Laird, DL. Random-effects model, inverse-variance weighting based on the DL method; OPMD, oral potentially malignant disorder. A RR > 1 suggests that podoplanin overexpression is associated with a higher malignant transformation risk. Diamonds indicate the pooled RR with their corresponding 95% CIs.

3.2 Subgroup analysis by type of OPMD

Figure S2. Forest plot graphically representing the stratified meta-analysis on the association between podoplanin overexpression and OPMDs malignant transformation risk by type of OPMD.

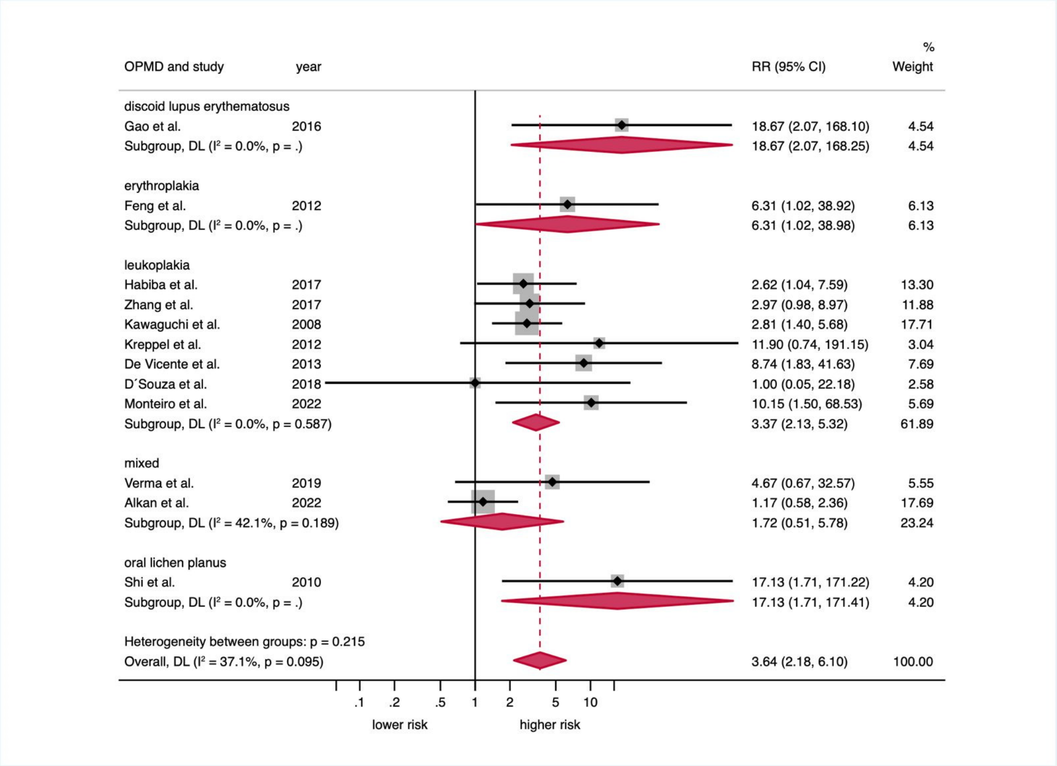

RR, relative risk; CI, confidence intervals, DerSimonian and Laird, DL; nr, not reported. Random-effects model, inverse-variance weighting based on the DL method; OPMD, oral potentially malignant disorder. A RR > 1 suggests that podoplanin overexpression is associated with a higher malignant transformation risk. Diamonds indicate the pooled RR with their corresponding 95% CIs.

3.3 Subgroup analysis by immunohistochemical pattern

Figure S3. Forest plot graphically representing the stratified meta-analysis on the association between podoplanin overexpression and OPMDs malignant transformation risk by immunohistochemical pattern.

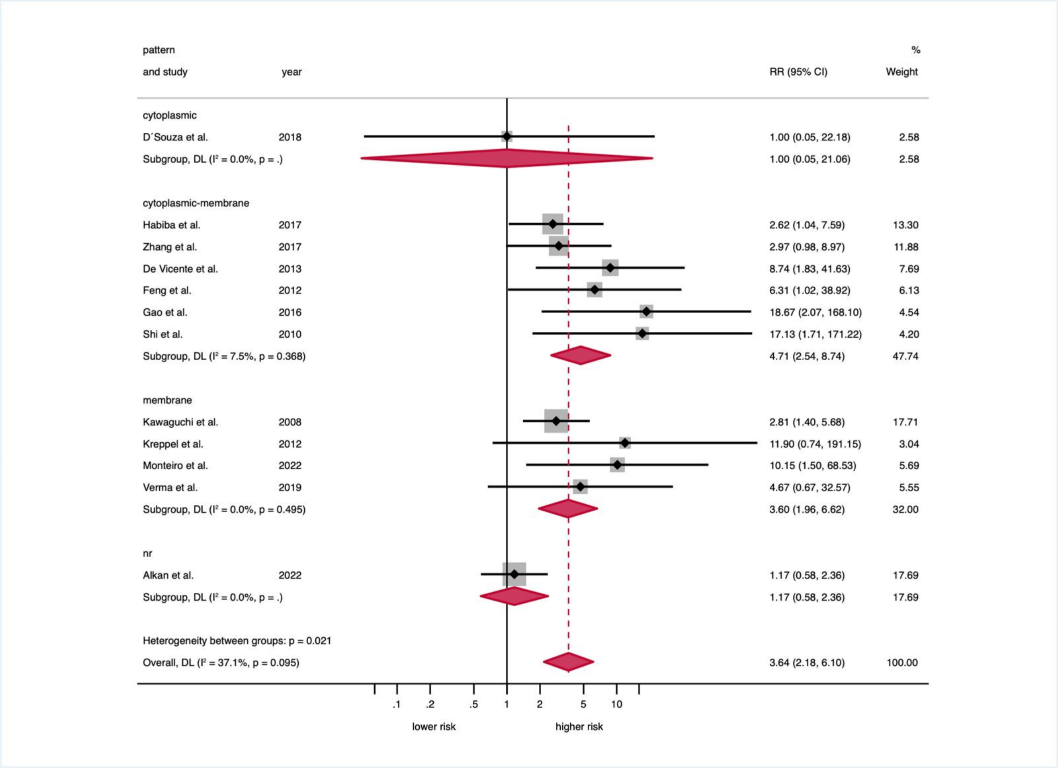

RR, relative risk; CI, confidence intervals, DerSimonian and Laird, DL. Random-effects model, inverse-variance weighting based on the DL method; na, not applicable; OPMD, oral potentially malignant disorder. A RR > 1 suggests that podoplanin overexpression is associated with a higher malignant transformation risk. Diamonds indicate the pooled RR with their corresponding 95% CIs.

3.4 Subgroup analysis by anti-podoplanin antibody

Figure S4. Forest plot graphically representing the stratified meta-analysis on the association between podoplanin overexpression and OPMDs malignant transformation risk by anti-podoplanin antibody.

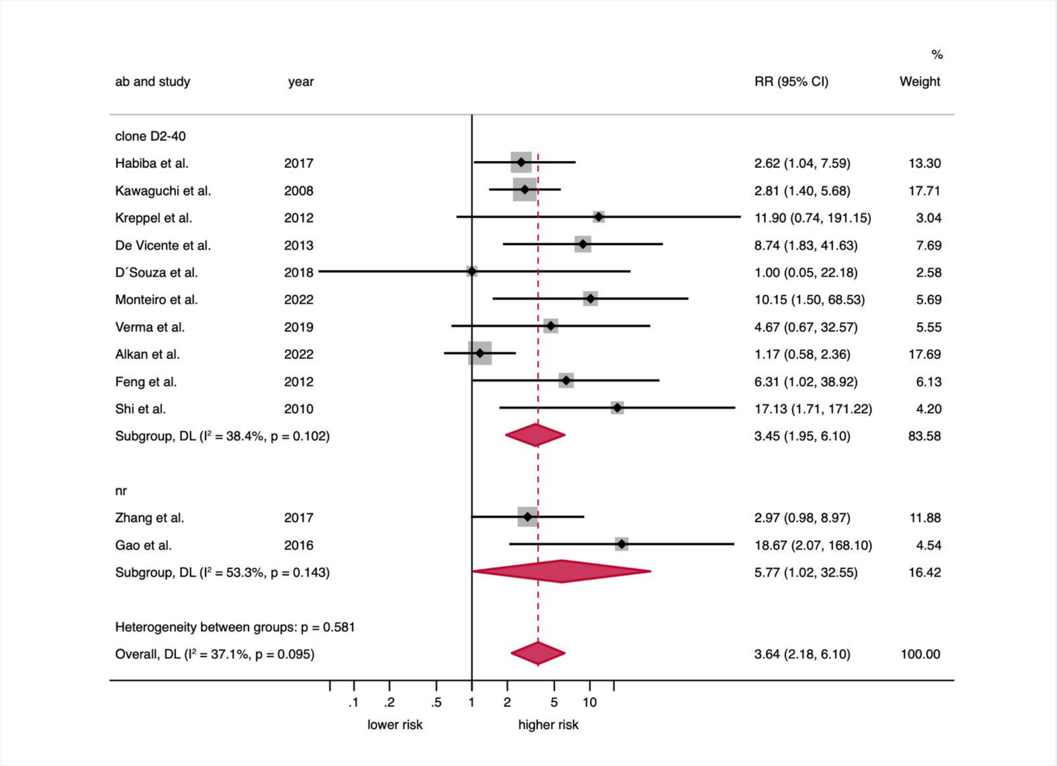

RR, relative risk; CI, confidence intervals, DerSimonian and Laird, DL; nr, not reported; na, not applicable. Random-effects model, inverse-variance weighting based on the DL method; OPMD, oral potentially malignant disorder. A RR > 1 suggests that podoplanin overexpression is associated with a higher malignant transformation risk. Diamonds indicate the pooled RR with their corresponding 95% CIs.

3.5 Subgroup analysis by anti-podoplanin antibody dilution

Figure S5. Forest plot graphically representing the stratified meta-analysis on the association between podoplanin overexpression and OPMDs malignant transformation risk by anti-podoplanin antibody dilution.

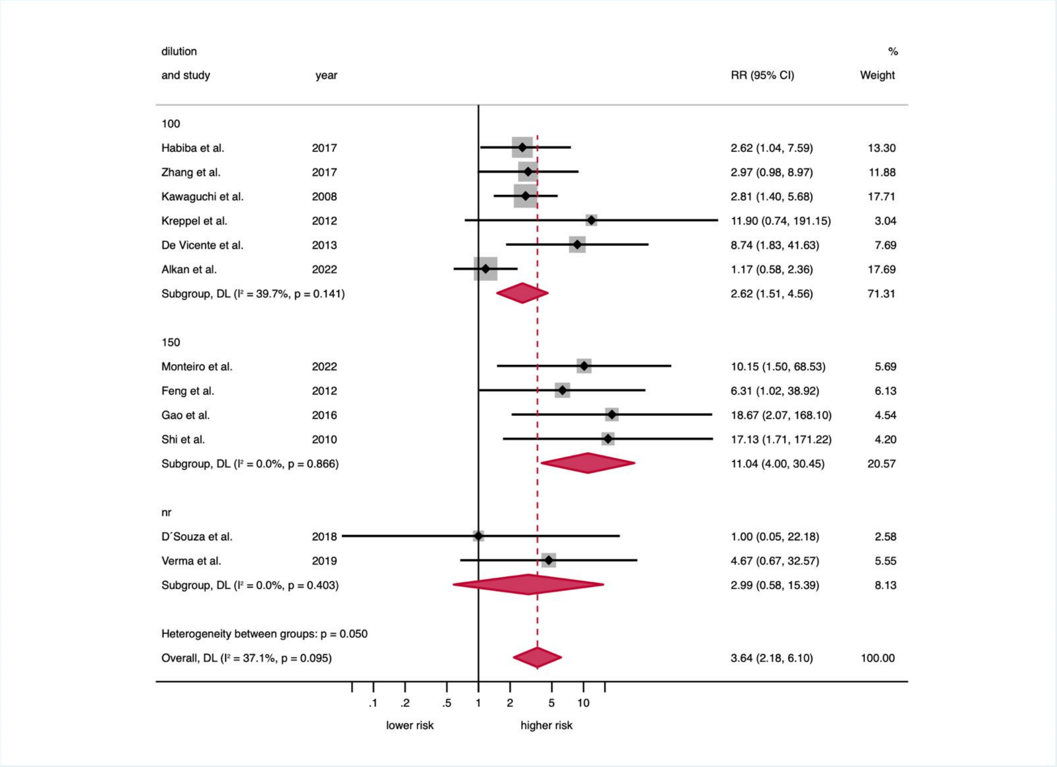

RR, relative risk; CI, confidence intervals, DerSimonian and Laird, DL; nr, not reported; na, not applicable. Random-effects model, inverse-variance weighting based on the DL method; OPMD, oral potentially malignant disorder. A RR > 1 suggests that podoplanin overexpression is associated with a higher malignant transformation risk. Diamonds indicate the pooled RR with their corresponding 95% CIs.

3.6 Subgroup analysis by anti-podoplanin antibody incubation time

Figure S6. Forest plot graphically representing the stratified meta-analysis on the association between podoplanin overexpression and OPMDs malignant transformation risk by anti-podoplanin antibody incubation time.

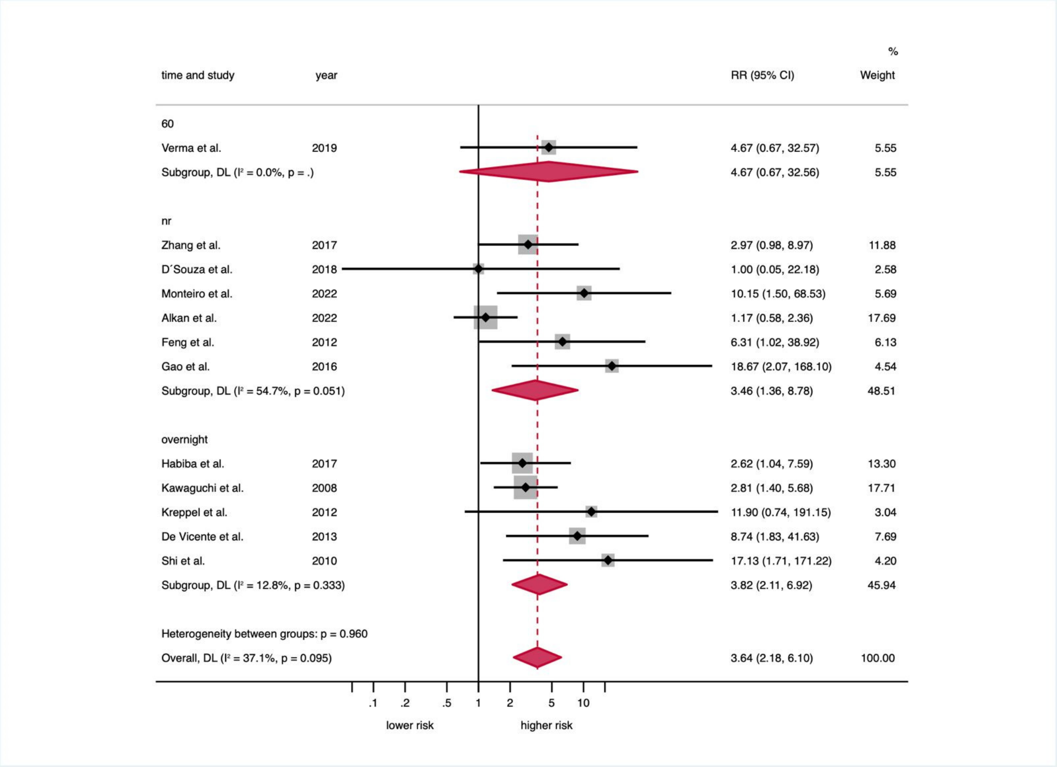

RR, relative risk; CI, confidence intervals, DerSimonian and Laird, DL; nr, not reported; na, not applicable. Random-effects model, inverse-variance weighting based on the DL method; OPMD, oral potentially malignant disorder. A RR > 1 suggests that podoplanin overexpression is associated with a higher malignant transformation risk. Diamonds indicate the pooled RR with their corresponding 95% CIs.

3.7 Subgroup analysis by anti-podoplanin antibody incubation temperature

Figure S7. Forest plot graphically representing the stratified meta-analysis on the association between podoplanin overexpression and OPMDs malignant transformation risk by anti-podoplanin antibody incubation temperature.

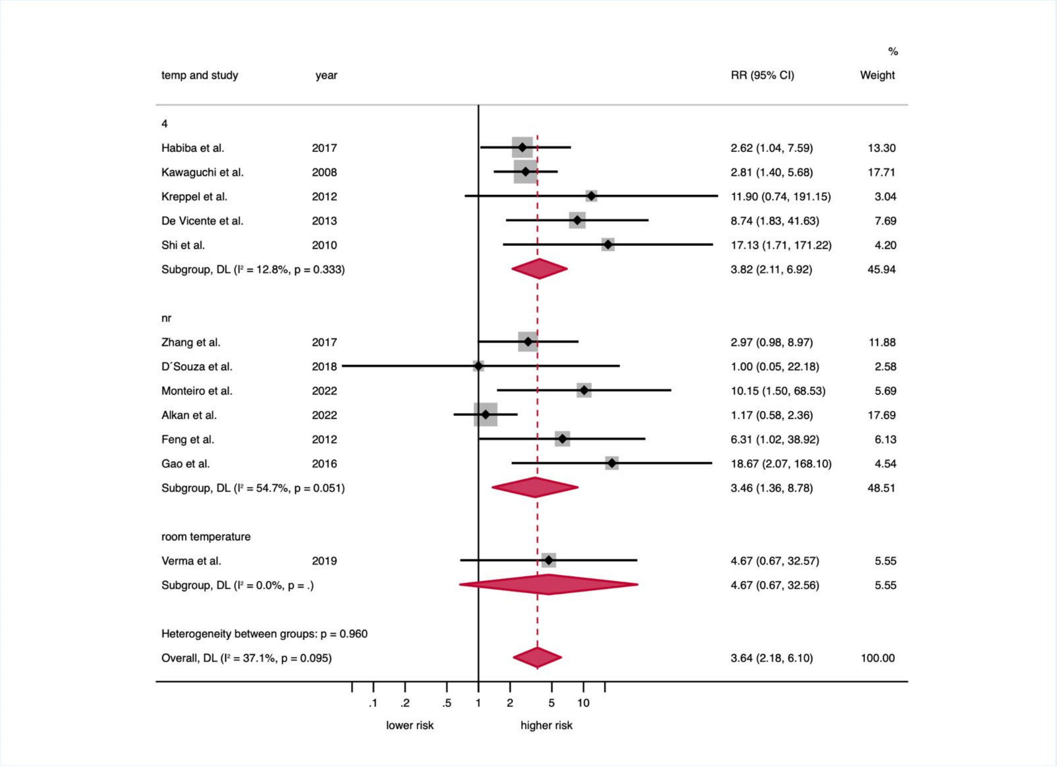

RR, relative risk; CI, confidence intervals, DerSimonian and Laird, DL; nr, not reported; na, not applicable. Random-effects model, inverse-variance weighting based on the DL method; OPMD, oral potentially malignant disorder. A RR > 1 suggests that podoplanin overexpression is associated with a higher malignant transformation risk. Diamonds indicate the pooled RR with their corresponding 95% CIs.

3.8 Subgroup analysis by cutoff point for podoplanin overexpression

Figure S8. Forest plot graphically representing the stratified meta-analysis on the association between podoplanin overexpression and OPMDs malignant transformation risk by cutoff point for podoplanin overexpression.

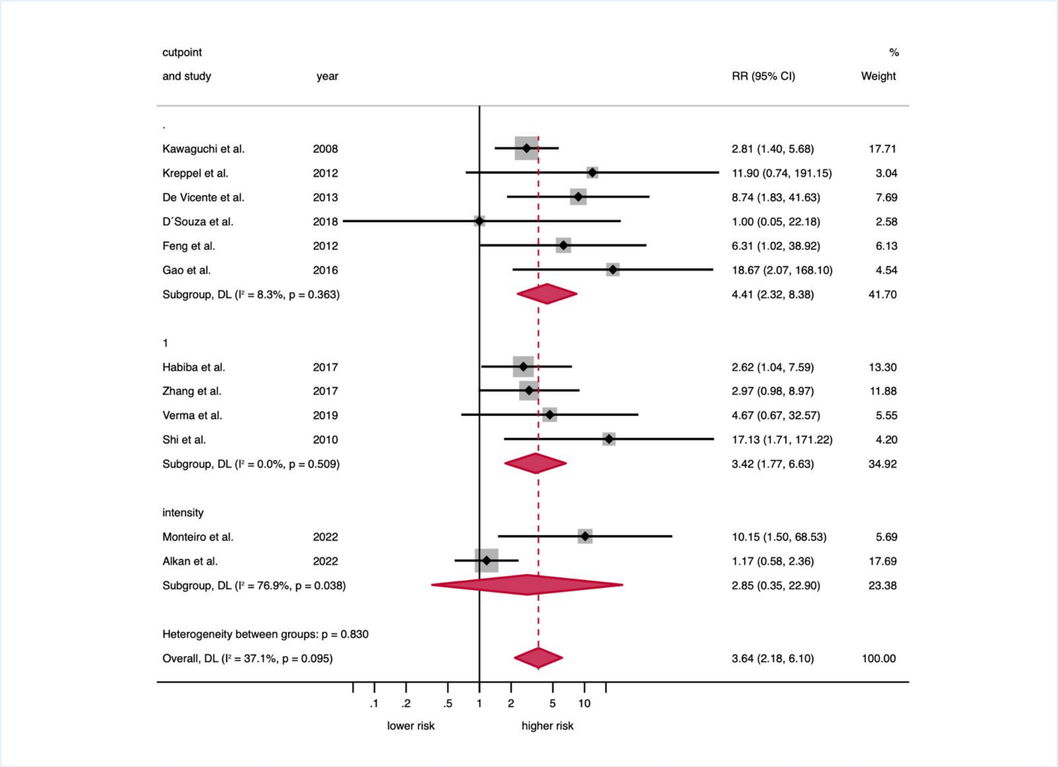

RR, relative risk; CI, confidence intervals, DerSimonian and Laird, DL; nr, not reported; na, not applicable. Random-effects model, inverse-variance weighting based on the DL method; OPMD, oral potentially malignant disorder. A RR > 1 suggests that podoplanin overexpression is associated with a higher malignant transformation risk. Diamonds indicate the pooled RR with their corresponding 95% CIs.

3.9 Subgroup analysis by overall risk of bias in primary-level studies

Figure S9. Forest plot graphically representing the stratified meta-analysis on the association between podoplanin overexpression and OPMDs malignant transformation risk by overall risk of bias in primary-level studies.

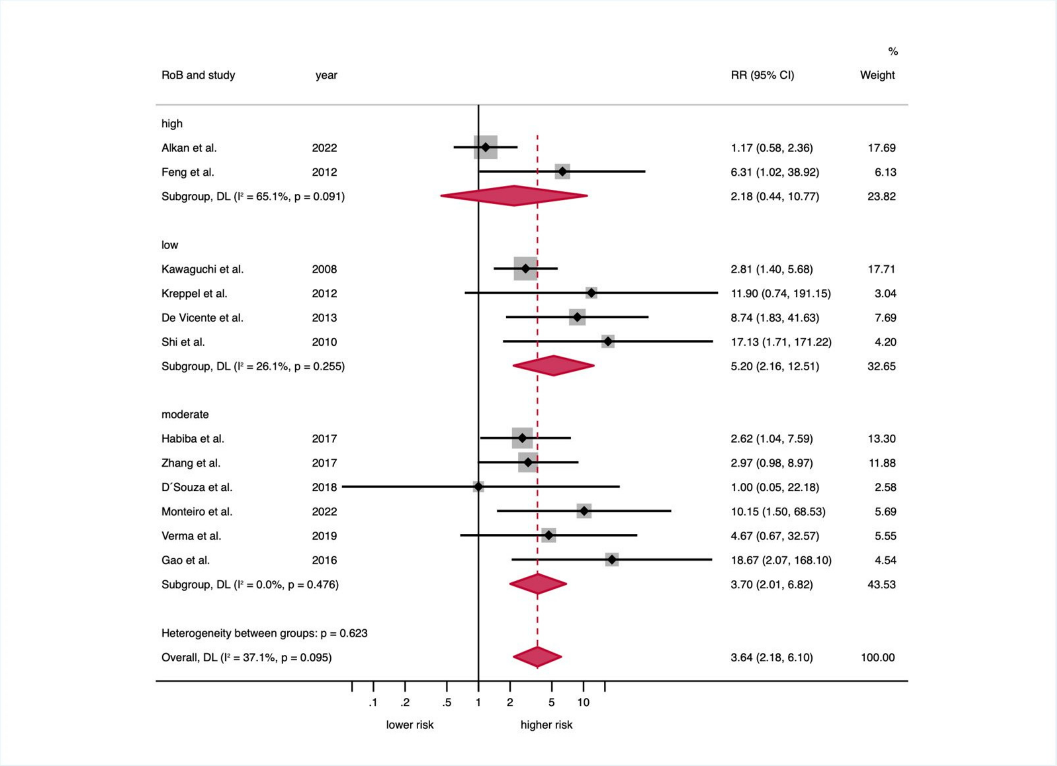

RR, relative risk; CI, confidence intervals, DerSimonian and Laird, DL. Random-effects model, inverse-variance weighting based on the DL method; OPMD, oral potentially malignant disorder. A RR > 1 suggests that podoplanin overexpression is associated with a higher malignant transformation risk. Diamonds indicate the pooled RR with their corresponding 95% CIs.

4. Analysis of small-study effects

Figure S10. A funnel plot of estimated effect size (logRR) against their SEs, graphically representing the analysis of small-study effects on the association between association between podoplanin overexpression and OPMDs malignant transformation risk. The red vertical line corresponds to the pooled effect size estimated in the meta-analysis. The two diagonal grey lines represent the pseudo-95%CIs. The blue circles represent the estimates from primary-level studies. The orange circles represent the identification of missing studies through trim-&-fill method.

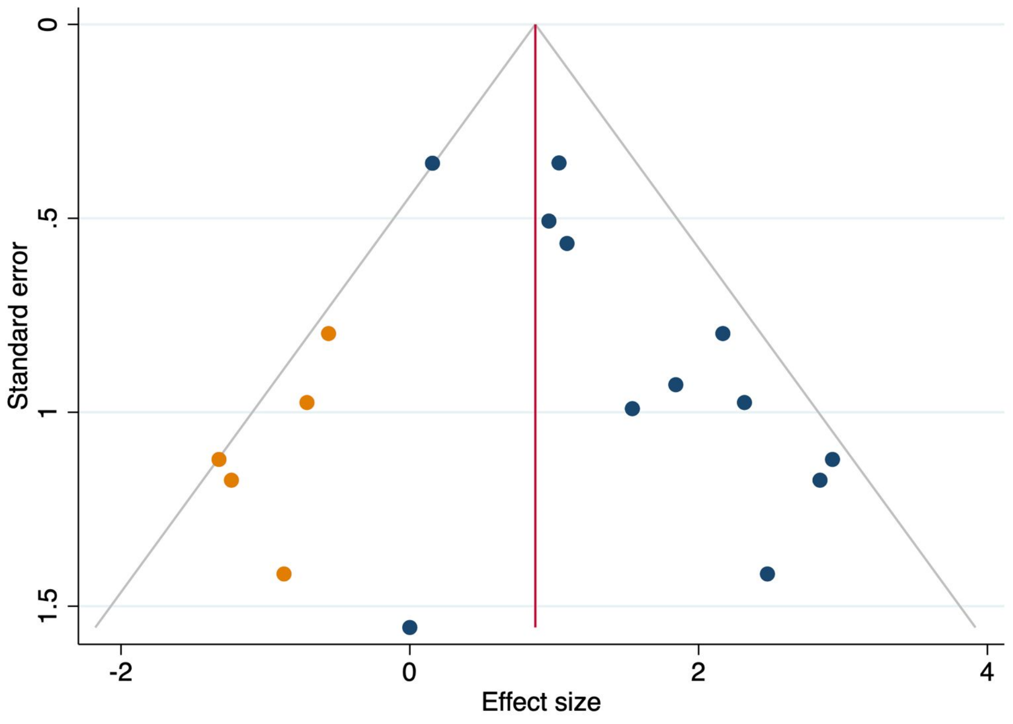

Abbreviations: log, natural logarithm (i.e., log base e); RR, relative risk; SE, standard error; CI, confidence intervals; OPMD, oral potentially malignant disorder.

5. Sensitivity analysis (leave-one-out method).

Figure S11. Interval plot graphically representing the sensitivity analysis of the studies pooled in the meta-analysis on the association between podoplanin overexpression and OPMDs malignant transformation risk. RR, relative risk; CI, confidence intervals; OPMD, oral potentially malignant disorder.

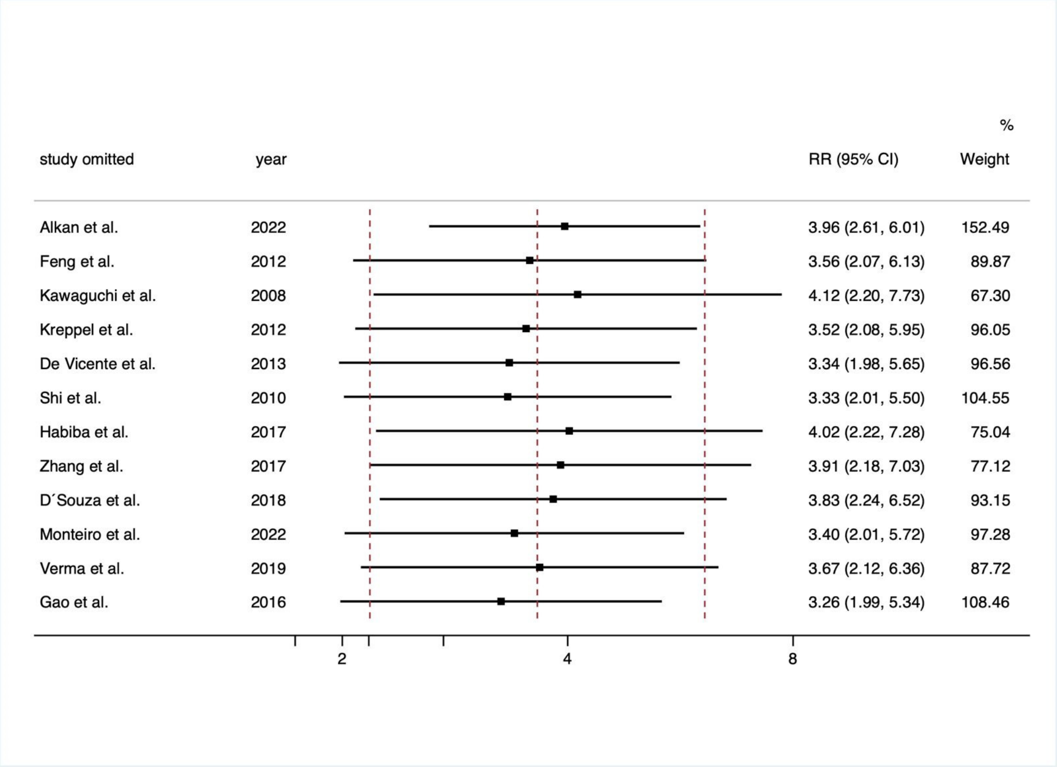

Sensitivity analysis ("leave-one-out" method) of the meta-analysis results, sequentially omitting one study at a time to investigate its influence on the overall result. In the interval plot, the usual diamond shape representing the pooled effect was replaced by vertical intermittent red lines, allowing a visual inspection analysis of influence.

6. List S1. List of full-text excluded studies, with reasons

Cross-sectional (n = 9)

Cheng SY, Shi K, Bai XR, Wu QW, Lv XQ. Double-staining of E-cadherin and podoplanin offer help in the pathological diagnosis of indecisive early-invasive oral squamous cell carcinoma. Int J Clin Exp Pathol. 2018 Jan 1;11(1):38-47. PMID: 31938085; PMCID: PMC6957954.

Deepa AG, Janardanan-Nair B, Varun BR. Podoplanin expression in oral potentially malignant disorders and oral squamous cell carcinoma. *J Clin Exp Dent*. 2017;9(12):e1418-e1424. Published 2017 Dec 1. doi:10.4317/jced.54213.

Funayama A, Cheng J, Maruyama S, et al. Enhanced expression of podoplanin in oral carcinomas in situ and squamous cell carcinomas. *Pathobiology*. 2011;78(3):171-180. doi:10.1159/000324926.

Gissi DB, Gabusi A, Tarsitano A, Luccarini L, Morandi L, Montebugnoli L. Podoplanin expression as a predictive marker of dysplasia in oral leukoplakia. *J Craniomaxillofac Surg*. 2018;46(5):759-764. doi:10.1016/j.jcms.2018.02.016.

Grochau KJ, Safi AF, Drebber U, Grandoch A, Zöller JE, Kreppel M. Podoplanin expression in oral leukoplakia—a prospective study. *J Craniomaxillofac Surg*. 2019;47(3):505-509. doi:10.1016/j.jcms.2018.12.005.

Inoue H, Miyazaki Y, Kikuchi K, et al. Podoplanin expression during dysplasia-carcinoma sequence in the oral cavity. *Tumour Biol*. 2012;33(1):183-194. doi:10.1007/s13277-011-0261-7.

Logeswari J, Malathi N, Thamizhchelvan H, Sangeetha N, Nirmala SV. Expression of podoplanin in oral premalignant and malignant lesions and its potential as a biomarker. *Indian J Dent Res*. 2014;25(3):305-310. doi:10.4103/0970-9290.138321.

Patil A, Patil K, Tupsakhare S, Gabhane M, Sonune S, Kandalgaonkar S. Evaluation of Podoplanin in Oral Leukoplakia and Oral Squamous Cell Carcinoma. *Scientifica (Cairo)*. 2015;2015:135298. doi:10.1155/2015/135298.

Shimamura Y, Abe T, Nakahira M, Yoda T, Murata S, Sugasawa M. Immunohistochemical analysis of oral dysplasia: diagnostic assessment by fascin and podoplanin expression. *Acta Histochem Cytochem*. 2011;44(6):239-245. doi:10.1267/ahc.11032.

Lack of essential data (n = 4)

Aiswarya A, Suresh R, Janardhanan M, Savithri V, Aravind T, Mathew L. An immunohistochemical evaluation of podoplanin expression in oral leukoplakia and oral squamous cell carcinoma to explore its potential to be used as a predictor for malignant transformation. *J Oral Maxillofac Pathol*. 2019;23(1):159. doi:10.4103/jomfp.JOMFP_272_17.

Mei Y, Zhang P, Zuo H, et al. Ebp1 activates podoplanin expression and contributes to oral tumorigenesis. *Oncogene*. 2014;33(29):3839-3850. doi:10.1038/onc.2013.354.

Saito H, Oikawa M, Kouketsu A, Takahashi T, Kumamoto H. Immunohistochemical assessment of Eph/ephrin expression in oral squamous cell carcinoma and precursor lesions. *Odontology*. 2020;108(2):166-173. doi:10.1007/s10266-019-00466-y.

Sundberg J, Pandey S, Giglio D, et al. Expression of p53, p63, podoplanin and Ki-67 in recurring versus non-recurring oral leukoplakia. *Sci Rep*. 2021;11(1):20781. Published 2021 Oct 21. doi:10.1038/s41598-021-99326-5.

Overlapping population (n = 2)

Habiba U, Kitamura T, Yanagawa-Matsuda A, Higashino F, Hida K, Totsuka Y, Shindoh M. HuR and podoplanin expression is associated with a high risk of malignant transformation in patients with oral preneoplastic lesions. Oncol Lett. 2016 Nov;12(5):3199-3207. doi: 10.3892/ol.2016.5061. Epub 2016 Aug 29. PMID: 27899983; PMCID: PMC5103919.

Saintigny P, El-Naggar AK, Papadimitrakopoulou V, Ren H, Fan YH, Feng L, Lee JJ, Kim ES, Hong WK, Lippman SM, Mao L. DeltaNp63 overexpression, alone and in combination with other biomarkers, predicts the development of oral cancer in patients with leukoplakia. Clin Cancer Res. 2009 Oct 1;15(19):6284-91. doi: 10.1158/1078-0432.CCR-09-0498. Epub 2009 Sep 22. PMID: 19773378; PMCID: PMC2756317.

Letter (n = 1)

Yang X, Shi L, Zhou Z, Liu W. Podoplanin and ABCG2 expression in oral erythroplakia revisited: Potential evidence for cancer stem cells driving the process of field cancerization. Oral Oncol. 2020 Feb;101:104368. doi: 10.1016/j.oraloncology.2019.07.011. Epub 2019 Jul 10. PMID: 31300272
